# Depletion of *Mycobacterium tuberculosis* transmembrane protein Rv3737 reduces pathogen survival and induces M1 macrophage polarization against tuberculosis

**DOI:** 10.3389/fcimb.2025.1592296

**Published:** 2025-09-02

**Authors:** Zhangli Peng, Chao Xu, Taixian You, Chengjie Shu, Qing Li, Nana Li, Renzhong He, Ling Chen, Lin Xu

**Affiliations:** ^1^ Tuberculosis Division of Pulmonary and Critical Care Medicine, Affiliated Hospital of Zunyi Medical University, Zunyi, Guizhou, China; ^2^ Department of General Practice, Affiliated Hospital of Zunyi Medical University, Zunyi, Guizhou, China; ^3^ Department of Immunology, Zunyi Medical University, Zunyi, Guizhou, China

**Keywords:** *Mycobacterium tuberculosis*, transmembrane protein, Rv3737, macrophage polarization, intracellular survival

## Abstract

**Objectives:**

*Mycobacterium tuberculosis* (Mtb) modulates macrophage polarization to evade host immunity and enhance intracellular survival. Rv3737, a probable conserved transmembrane protein in Mtb, has an unclear biological function. This study investigates the role of Rv3737 in regulating macrophage polarization and Mtb survival within host cells.

**Methods:**

The structure of Rv3737 was predicted using bioinformatics tools. Macrophage polarization markers were assessed by real-time PCR for M1/M2-associated cytokines, and flow cytometry for CD86^+^/CD206^+^ expression. RNA sequencing, along with KEGG and GO analyses, was used to explore underlying regulatory pathways. Western blotting evaluated the phosphorylation status of NF-κB (P65, IκB) and MAPK (ERK, P38, JNK) signaling components. Colony-forming units (CFUs) and inducible nitric oxide synthase (iNOS) levels were examined in H37RvΔRv3737-infected macrophages pretreated with specific inhibitors (JSH-23, U0126-EtOH, SB203580, SP600125).

**Results:**

Rv3737 is predicted to contain 10 transmembrane segments enriched in aliphatic amino acids. Deletion of Rv3737 in H37Rv (H37RvΔRv3737) led to upregulation of M1 markers (TNF-α, IL-1β, IL-6, iNOS, MCP-1, CD86) and downregulation of M2 markers (Arg-1, IL-10, TGF-β, CD206). Conversely, overexpression of Rv3737 (MS_Rv3737) promoted M2 polarization. RNA sequencing indicated NF-κB pathway activation in macrophages infected with H37RvΔRv3737, along with increased phosphorylation of P65, IκB, ERK, and P38. Inhibition of NF-κB (with JSH-23) and P38 MAPK (with SB203580) reduced iNOS levels and partially restored Mtb survival, indicating that Rv3737 deletion enhances the macrophage antimicrobial response.

**Conclusions:**

Rv3737 suppresses M1 macrophage polarization to promote Mtb survival. Its deletion enhances host antimicrobial activity by activating NF-κB and MAPK signaling pathways. Targeting Rv3737 may represent a novel strategy for tuberculosis therapy.

## 1 Introduction

Tuberculosis (TB), caused by *Mycobacterium tuberculosis* (Mtb), remains a significant global health threat. In 2022, approximately 10.6 million individuals developed TB, and 1.3 million died from the disease—marking the first increase in TB-related mortality among HIV-negative individuals in the past 15 years (WHO, 2023). Macrophages, as the first line of defense, play a central role in the host immune response by phagocytosing Mtb. However, Mtb has evolved sophisticated mechanisms to evade immune surveillance and persist within host cells (Zarin et al., 2024). The complex interaction between Mtb and the host immune system largely determines the course and outcome of infection. Therefore, elucidating the immune mechanisms underlying TB pathogenesis is essential for developing effective treatment strategies (Hmama et al., 2015).

One of the key immune evasion strategies employed by Mtb is the manipulation of macrophage polarization (Pham and Monack, 2023). Upon infection, Mtb modulates macrophage polarization to facilitate its survival and dissemination. Macrophages exhibit plasticity and can polarize into either classically activated M1 or alternatively activated M2 phenotypes (Zheng et al., 2024). M1 macrophages produce pro-inflammatory cytokines such as iNOS, TNF-α, IL-1β, IL-6, and IL-12b, which promote Th1 responses and possess bactericidal activity. In contrast, M2 macrophages secrete anti-inflammatory cytokines like IL-10 and TGF-β and are involved in tissue repair and immune regulation (Zhang et al., 2024). The polarization state of macrophages is dynamic and reversible, depending on environmental cues (Wang et al., 2014). Numerous Mtb-derived factors, including secreted and membrane-associated proteins, contribute to the modulation of macrophage polarization and aid in immune evasion. Several secreted proteins, such as Rv1987 (Liu et al., 2023), PPE36 (Gong et al., 2021), and HSP16.3, are known to influence macrophage phenotypes. For example, ESAT-6 promotes M1 polarization early in infection but later skews macrophages toward an M2-like state (Sun et al., 2024). Rv2882c has been shown to regulate macrophage polarization (Choi et al., 2016). In addition to secreted proteins, transmembrane proteins of Mtb have also been implicated in modulating macrophage polarization. For example, the transmembrane protein Rv0309 may inhibit M1 polarization (Peng et al., 2022), while Rv0426c has been reported to promote Mtb survival by influencing macrophage polarization (Ruan et al., 2020). Understanding the paradoxical roles of macrophage polarization is essential for uncovering the underlying mechanisms. However, whether other Mtb transmembrane proteins are involved in regulating macrophage polarization—and the specific pathways by which they exert their effects—remains largely unclear.

Rv3737, a transmembrane protein of unknown function, has recently garnered attention for its potential role in Mtb pathogenesis. Structural predictions suggest Rv3737 is encoded by a non-essential gene in the H37Rv genome (Minato et al., 2019), and it shares 37.25% sequence homology with the threonine efflux carrier ThrE from *Corynebacterium glutamicum*. It contains three ThrE structural domains and is predicted to function in amino acid transport. Preliminary findings indicate that deletion of Rv3737 disrupts threonine metabolism and upregulates the expression of inflammatory cytokines such as TNF-α, IL-1β, and IL-6 (Li et al., 2022). Moreover, Rv3737 expression has been linked to amikacin resistance (Li et al., 2024), suggesting a possible association with disease severity in TB patients. Despite these observations, the role of Rv3737 in macrophage polarization and immune regulation remains largely unexplored.

In this study, we investigated the effect of Rv3737 on macrophage polarization and its influence on Mtb intracellular survival. Using an Rv3737-deletion strain (H37RvΔRv3737) and an Rv3737-overexpressing strain (MS_Rv3737), we explored how this protein modulates host immune responses. Our findings demonstrate that Rv3737 is a transmembrane protein whose deletion induces M1-type macrophage polarization, characterized by elevated expression of iNOS, TNF-α, IL-1β, IL-6, and CD86. Mechanistically, Rv3737 depletion activates the NF-κB and p38 MAPK signaling pathways, as evidenced by increased phosphorylation of p65 and p38. Pharmacological inhibition of these pathways reversed the polarization phenotype, reduced iNOS expression, and restored Mtb survival within macrophages. Together, these results reveal that Rv3737 plays a critical role in modulating macrophage polarization and suggest that it may serve as a potential therapeutic target for enhancing host immunity against TB.

## 2 Materials and methods

### 2.1 Bacterial strains, cell lines, and reagents

The *Mycobacterium tuberculosis* H37Rv wild-type strain (H37Rv-WT) was generously provided by the Tuberculosis Control Institute of Guizhou CDC. *Mycobacterium smegmatis* MC2155 was obtained from Zunyi Medical University. Recombinant strains—including MS_Vec (vector control), MS_Rv3737 (Rv3737-overexpressing), and H37RvΔRv3737 (Rv3737-deleted)—were constructed in our previous study. All strains were cultured in Middlebrook 7H9 broth (BD Biosciences, USA) supplemented with 10% OADC and the appropriate antibiotics: kanamycin (SolarBio, China) and hygromycin B (Sigma, USA). RAW264.7 murine macrophage cells (ATCC) were maintained in Dulbecco’s Modified Eagle Medium (DMEM; Gibco, USA) supplemented with 10% fetal bovine serum (FBS) and 1% penicillin-streptomycin. Cells were cultured at 37°C in a humidified atmosphere containing 5% CO_2_.

### 2.2 Mycobacteria infected macrophages and mice

RAW264.7 cells were seeded in six-well plates and allowed to adhere overnight at 37°C. The following day, cells were infected with either MS_Vec, MS_Rv3737, H37Rv-WT, or H37RvΔRv3737 at a multiplicity of infection (MOI) of 10. At designated time points post-infection, culture supernatants were collected for cytokine analysis. Cells were washed three times with phosphate-buffered saline (PBS) and then lysed for downstream analyses, including gene expression, protein detection, or bacterial load determination.

6–8 weeks C57BL/6 mice were purchased from the Hunan Slack Jingda Experimental Animal Co., Ltd. All animal experiments and protocols were approved by the Ethics Committee of Zunyi Medical University (ZMU22-2302-136). The mice were housed according to the standard humane animal husbandry protocol. Each mouse was intranasally infected with MS_Vec and MS_Rv3737 strains at 2×10^7^/ml. After six days of infection, the Lung tissues were harvested from euthanized mice and promptly immersed in cold PBS. The tissues were mechanically homogenized with a tissue homogenizer in RIPA lysis buffer augmented with protease and phosphatase inhibitors. The homogenates underwent centrifugation at 12,000 × g for 10 minutes at 4°C to eliminate tissue debris. The resultant supernatants were collected and preserved at −80 °C until needed. Protein concentrations were quantified via a BCA assay (Thermo Fisher Scientific, 23225). The supernatants were subsequently utilized for Western blot investigation of target proteins. Lung tissues were homogenized and then diluted with PBS. 20 μL of tissue homogenate was added to 7H10 plates with or without kanamycin to analyze the CFU. A few of the lung tissue samples were fixed in 10% formalin and then embedded in paraffin, followed by hematoxylin and eosin staining and acid-fast staining for evaluating the pathological changes in the lung tissue.

### 2.3 Intracellular survival of H37Rv△Rv3737 and H37Rv-WT strains: colony-forming unit assay

RAW264.7 cells were infected with *M. tuberculosis* H37Rv-WT or H37RvΔRv3737 strains at a multiplicity of infection (MOI) of 10. Four hours post-infection, the culture medium was removed, and cells were washed thoroughly with PBS to eliminate non-internalized bacteria. Cells were then incubated in 10% DMEM containing 200 µg/mL amikacin and 1% antibiotic-antimycotic solution (Gibco) for an additional 24 or 48 hours to kill any remaining extracellular bacteria. To assess intracellular Mtb survival, cells were lysed with 0.01% Triton X-100. The resulting lysates were serially diluted (10^-^¹ and 10^-^²), plated in triplicate on Middlebrook 7H10 agar, and incubated at 37°C for 3–4 weeks. Colony-forming units (CFUs) were then counted to determine bacterial burden.

### 2.4 Real-time quantitative PCR analysis of M1/M2-associated cytokine mRNA expression

Total RNA was extracted from macrophages using RNAiso PLUS reagent, and cDNA was synthesized using the PrimeScript RT reagent kit, following previously described protocols (Zheng et al., 2024). Quantitative real-time PCR (qRT-PCR) was performed using specific primers (listed in **Supplementary Table 1**). Gene expression levels were normalized to GAPDH and calculated using the 2^-△△CT^ method.

### 2.5 Flow cytometry analysis of CD86^+^/CD206^+^ expression in MS_Rv3737-infected macrophages

RAW264.7 cells (5–10 × 10^5^ cells/mL) were seeded in six-well plates and infected with MS_Vec or MS_Rv3737 for 12 or 24 hours. After incubation, cells were blocked with 0.25 µg of Fc receptor blocking buffer on ice for 5–10 minutes in the dark. Surface staining was performed using anti-mouse Percp-CD11b (BioLegend, 101229) and anti-mouse APC-CD86 (BioLegend, 159215) or the corresponding APC isotype control (APC Rat IgG2a, BioLegend, 400511) for 30 minutes in the dark. Cells were then washed three times with PBS and fixed with 500 µL of Fix/Perm Buffer for 30 minutes. After centrifugation at 1000 rpm for 5 minutes and removal of the supernatant, cells were permeabilized with Intracellular Staining Perm/Wash Buffer. Intracellular staining was performed using anti-mouse PE-CD206 (BioLegend, 141705) or its isotype control (PE Rat IgG2a, BioLegend, 400507) for 30 minutes in the dark. Finally, cells were washed 2–3 times and resuspended in 300 µL of 1× PBS. Flow cytometry was performed using an Accuri C6 system (BD Biosciences, San Diego, CA, USA).

### 2.6 RNA sequencing, coupled with KEGG and GO analyses, explored the mechanisms underlying polarization

RAW264.7 cells were infected with either H37Rv-WT or H37RvΔRv3737 at an MOI of 10 for 24 hours. After infection, cells were washed with PBS, and total RNA was extracted using RNAiso Plus (Takara, 9108Q). RNA sequencing was performed by Novogene Bioinformatics Technology (Beijing, China) using the Illumina HiSeq X Ten platform with 150 bp paired-end reads. Sequencing quality was assessed using FASTQC (v0.11.3) to identify adaptor contamination and low-quality bases. Clean reads were aligned to the mouse reference genome using HISAT2. Differential gene expression analysis was performed using DESeq2 (v1.18.1) in R (v3.4.4), applying a fold-change cutoff >1.2 and p-value <0.05. Functional enrichment analysis of differentially expressed genes was conducted using Gene Ontology (GO) and Kyoto Encyclopedia of Genes and Genomes (KEGG) pathway databases.

### 2.7 Western blot assays of critical signaling molecule expression

RAW264.7 cells were infected with MS or H37Rv strains for 0, 2, or 4 hours. After three PBS washes, cells were lysed using RIPA buffer supplemented with a protease inhibitor cocktail (MCE, HY-K0010) and PMSF (Solarbio, P0100). Lysates were centrifuged at 12,000 rpm for 10 minutes, and the supernatants were collected. Protein concentrations were determined using a BCA Protein Assay Kit (Thermo Fisher Scientific, 23225). Proteins were separated by SDS-PAGE, transferred to PVDF membranes, and blocked with 5% non-fat milk for 1 hour at room temperature. Membranes were incubated overnight at 4°C with primary antibodies targeting: P65 (Proteintech, 66535-1-Ig), p-P65 (CST, 3033S), IκB (CST, 4812S), p-IκB (CST, 2859S), ERK (Proteintech, 67170-1-Ig), p-ERK (CST, 4370S), JNK (Proteintech, 511531-1-AP), p-JNK (Proteintech, 80024-1-RR), P38 (Proteintech, 66234-1-Ig), p-P38 (CST, 4511S), iNOS (CST, 2982S), Arg-1 (CST, 93668S), GAPDH (Proteintech, 60004-1-Ig), and Tubulin (Proteintech, 11224-1-AP). After washing, membranes were incubated with HRP-conjugated secondary antibodies—Goat Anti-Mouse IgG (SA00001-1) or Goat Anti-Rabbit IgG (SA00001-2)—for 2 hours at room temperature. Target proteins were visualized using ECL chemiluminescence (GE Healthcare) and quantified with ImageJ software.

### 2.8 Pharmacological inhibition assays: CFU and iNOS expression in inhibitor-pretreated macrophages

RAW264.7 cells were pretreated with one of the following inhibitors for 2 hours: 10 μM JNK inhibitor SP600125 (MCE, HY-12041), 10 μM P38 inhibitor SB203580 (MCE, HY-10256), 10 μM ERK inhibitor U0126-ETOH (MCE, HY-12031), or 30 μM P65 inhibitor JSH-23 (MCE, HY-13982). Following pretreatment, cells were infected with H37Rv-WT or H37RvΔRv3737 at an MOI of 10 for 24 hours. Total RNA was extracted as previously described, and iNOS mRNA levels were analyzed via qRT-PCR. For CFU assays, inhibitor-pretreated RAW264.7 cells were infected with H37Rv-WT or H37RvΔRv3737 for 4 hours. Intracellular bacterial survival was assessed via CFU counting as described in Section 2.3.

### 2.9 Structural prediction and bioinformatics analysis of Rv3737

Bioinformatics analysis of Rv3737 was conducted using several online tools. The FASTA sequence was retrieved from the National Center for Biotechnology Information (NCBI). Transmembrane domains were predicted using TMHMM (http://www.cbs.dtu.dk/services/TMHMM/), signal peptide regions using SignalP (http://www.cbs.dtu.dk/services/SignalP/), and conserved motifs via MEME Suite (http://meme-suite.org/).

### 2.10 Statistical analysis

All data are presented as mean ± standard deviation (SD). Statistical analysis was performed using GraphPad Prism 10.3 software. Comparisons between two groups were made using Student’s *t*-test, while comparisons across multiple groups were evaluated using two-way ANOVA. A *p*-value < 0.05 was considered statistically significant.

## 3 Results

### 3.1 Rv3737 is a transmembrane protein rich in aliphatic amino acids

**Figure 1:**
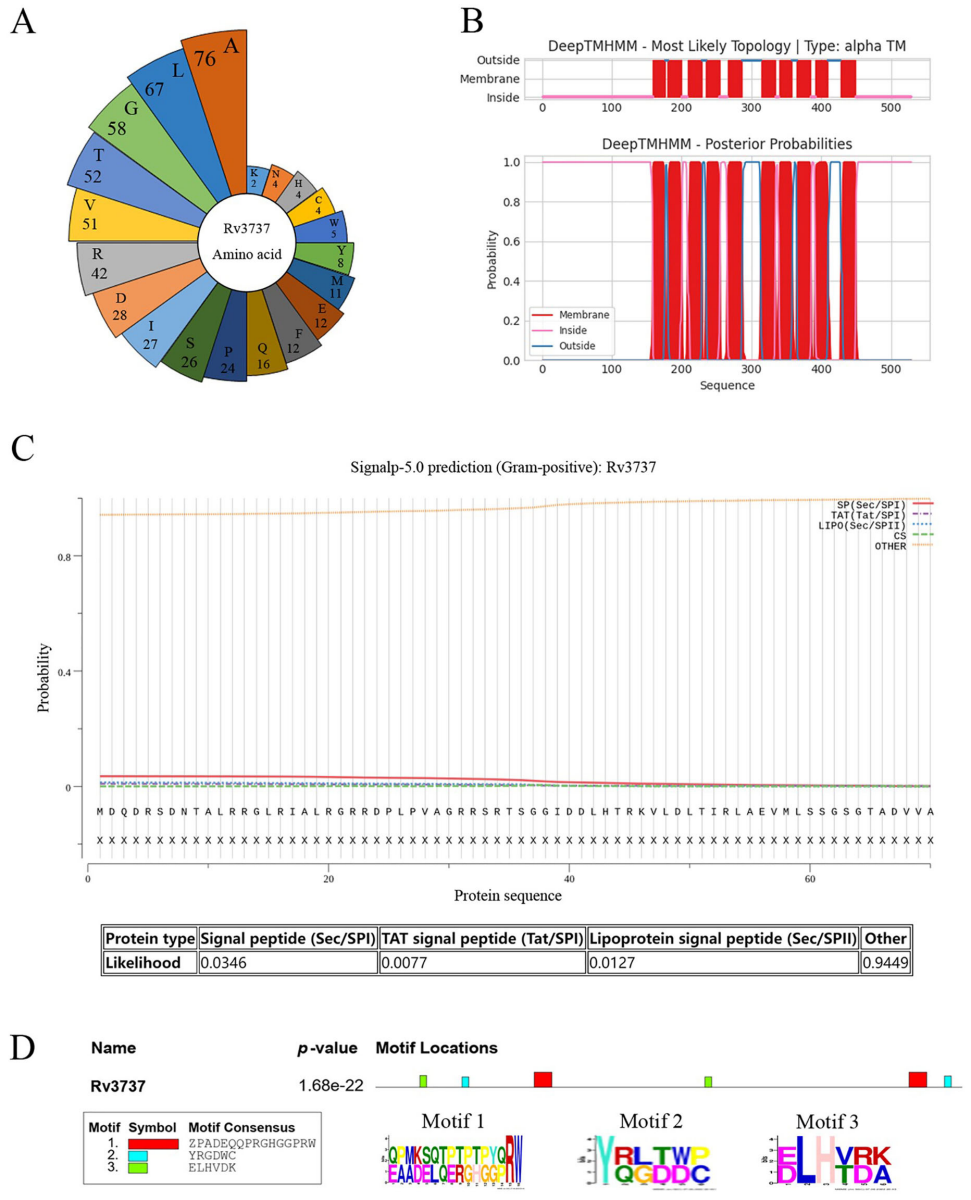
Rv3737 as a transmembrane protein rich in aliphatic amino acids. **(A)** Distribution and abundance of amino acids in the Rv3737 protein. **(B)** Prediction of transmembrane domains using TMHMM. **(C)** Signal peptide prediction of Rv3737. **(D)** Conservation of Rv3737 motifs across four virulent *Mycobacterium* strains, including *M. bovis* AF2122-97.

The structure and function of Rv3737 remain largely uncharacterized. To gain insights into its physicochemical properties, we conducted a comprehensive bioinformatics analysis. The five most abundant amino acids in Rv3737 were found to be aliphatic, contributing to a high aliphatic index of 111.63 (**Figure 1A**). Additionally, the calculated grand average of hydropathicity (GRAVY) was 0.45, suggesting strong lipophilicity and a propensity for interaction with lipid membranes. Transmembrane domain prediction revealed that Rv3737 contains 10 transmembrane segments (**Figure 1B**), supporting its classification as a transmembrane protein. Signal peptide prediction using SignalP 5.0 indicated that Rv3737 does not belong to the Sec/SPI, Sec/SPII, or Tat/SPI secretion pathways, implying it is a non-secretory membrane protein (**Figure 1C**). Sequence conservation analysis showed that Rv3737 is highly conserved in *Mycobacterium bovis* AF2122/97 (**Figure 1D**), suggesting a potentially important role in bacterial physiology or virulence. Taken together, these findings indicate that Rv3737 is a conserved, lipophilic transmembrane protein likely involved in essential mycobacterial functions.

### 3.2 Rv3737 suppresses M1 macrophage polarization and pro-inflammatory cytokine production

**Figure 2:**
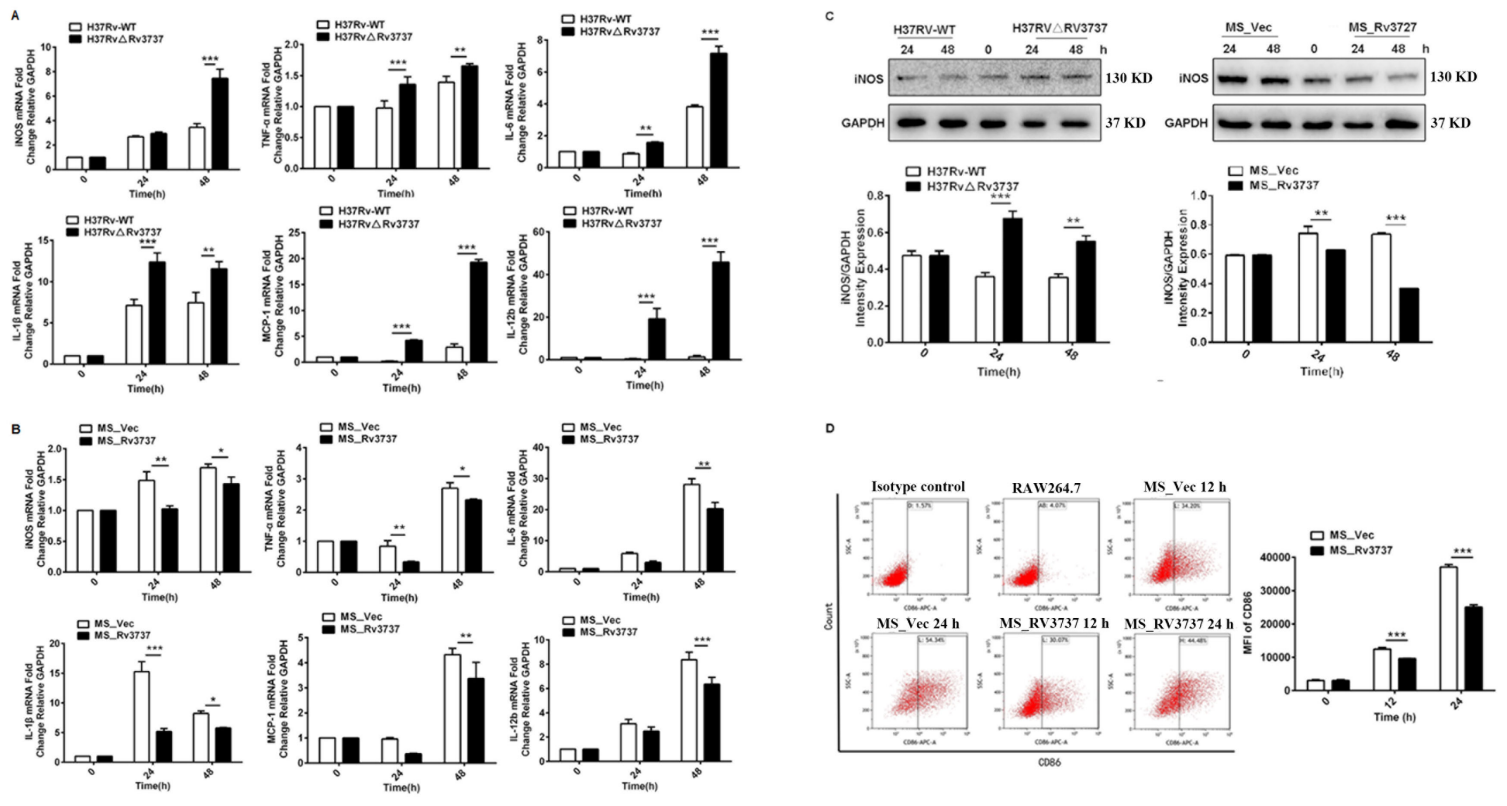
Rv3737 inhibits M1 macrophage polarization and pro-inflammatory cytokine expression. RAW264.7 cells were infected with H37Rv-WT, H37Rv△Rv3737, MS_Vec, or MS_Rv3737 at an MOI of 10 for 24 or 48 hours. **(A, B)** mRNA levels of M1 cytokines (TNF-α, IL-1β, IL-6, iNOS, MCP-1) assessed by real-time PCR. **(C)** Intracellular iNOS protein expression analyzed by Western blot. **(D)** Mean fluorescence intensity (MFI) of CD86, an M1 surface marker, measured by flow cytometry. *p < 0.05, **p < 0.01, ***p < 0.001.

Macrophage polarization is a dynamic process, and M1-type macrophages play a pivotal role in the immune response against intracellular pathogens (Thomas et al., 2024). To investigate the role of Rv3737 in macrophage polarization, we infected RAW264.7 cells with either H37Rv-WT or H37RvΔRv3737 strains. Deletion of Rv3737 significantly enhanced M1 macrophage polarization, as demonstrated by the upregulated mRNA expression of pro-inflammatory cytokines, including TNF-α, IL-1β, IL-6, MCP-1, and iNOS in H37RvΔRv3737-infected cells compared to H37Rv-WT-infected controls (**Figure 2A**). Conversely, overexpression of Rv3737 in MS_Rv3737 significantly suppressed the expression of these cytokines (**Figure 2B**). In line with these transcriptional changes, iNOS protein levels were markedly elevated in H37RvΔRv3737-infected macrophages (**Figure 2C**). Furthermore, the expression of CD86—a surface marker indicative of M1 polarization—was significantly reduced in MS_Rv3737-infected cells at both 12 and 24 hours, as measured by mean fluorescence intensity (MFI) (**Figure 2D**). To further investigate the Rv3737-mediated macrophage polarization *in vivo*, a mouse model of MS_Vec or Ms_Rv3737 was constructed via intranasal infection. MS_Rv3737 inhibited iNOS mRNA expression levels in the lung tissue compared with the MS_Vec-infected group (**Supplementary Figure 1C**). The western blotting results indicated that Ms_Rv3737 decreased the expression of iNOS in the lung tissue (**Supplementary Figures 1B, E**). These findings indicate that Rv3737 negatively regulates M1 macrophage polarization and pro-inflammatory cytokine release.

### 3.3 Rv3737 promotes M2 macrophage polarization and anti-inflammatory cytokine production

**Figure 3:**
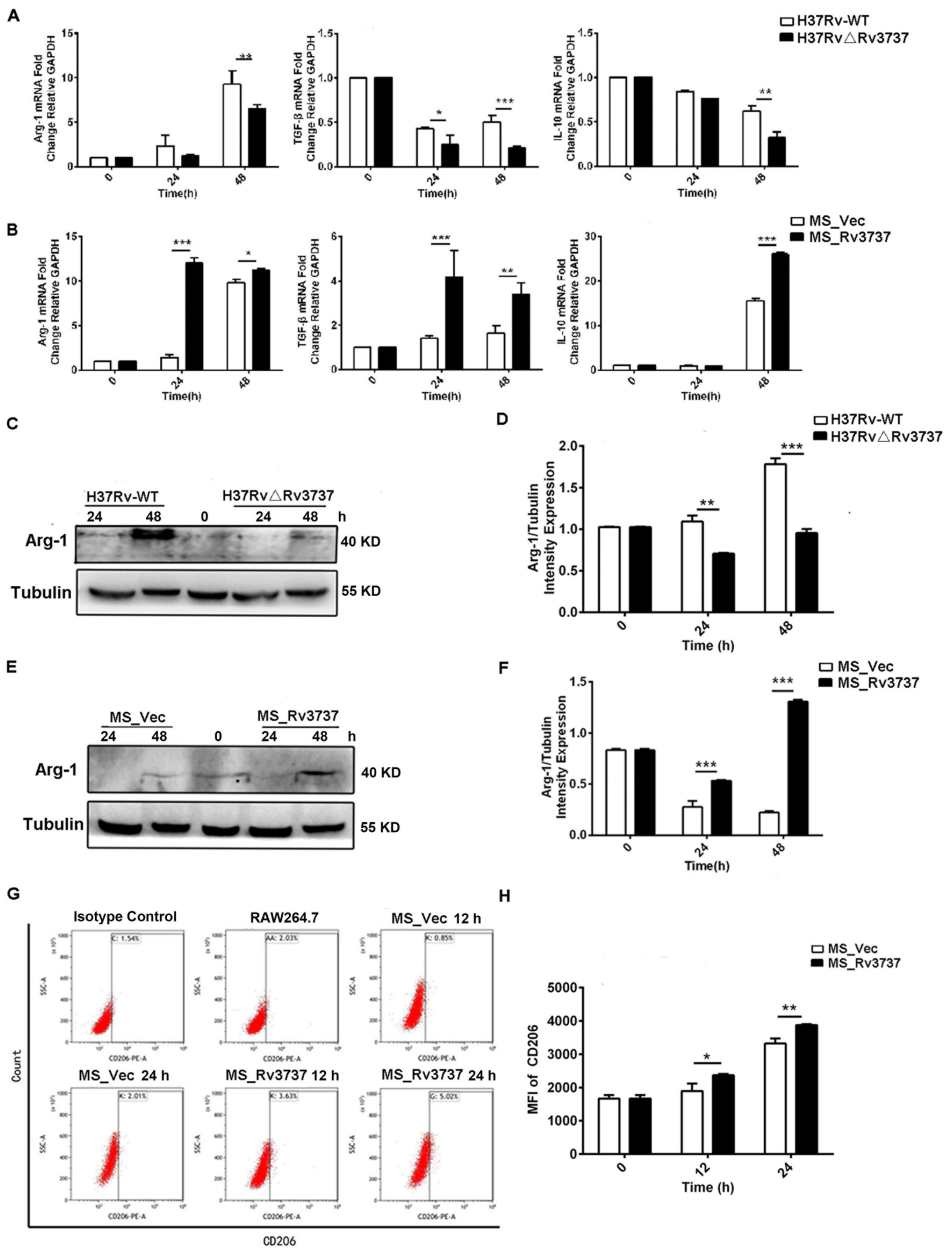
Rv3737 promotes M2 macrophage polarization and anti-inflammatory cytokine expression. RAW264.7 cells were infected as above. **(A)** mRNA levels of M2 cytokines (Arg-1, IL-10, TGF-β) in H37Rv△Rv3737- vs. H37Rv-WT-infected cells. **(B)** M2 cytokine expression in MS_Rv3737- vs. MS_Vec-infected cells. **(C, E)** Arg-1 protein expression assessed by Western blot. **(D, F)** Quantification of Arg-1 band intensity. **(G, H)** MFI of the M2 surface marker CD206 detected by flow cytometry. *p < 0.05, **p < 0.01, ***p < 0.001. Data are shown as mean ± SD from independent experiments.

M2 macrophages contribute to an immunosuppressive microenvironment that supports Mtb persistence and latency. To determine whether Rv3737 influences M2 polarization, we assessed the expression of M2-associated markers in infected RAW264.7 cells. Macrophages infected with H37RvΔRv3737 exhibited a significant reduction in the mRNA expression of Arg-1, TGF-β, and IL-10 compared to those infected with H37Rv-WT (**Figure 3A**). Arg-1 protein expression was also markedly decreased in H37RvΔRv3737-infected cells (**Figures 3C, D**). In contrast, overexpression of Rv3737 in MS_Rv3737-infected cells led to increased mRNA and protein expression of Arg-1, TGF-β, and IL-10 (**Figures 3B, E, F**). Consistently, *in vivo* experiments further confirmed that Rv3737 promotes Arg-1 expression, as evidenced by increased Arg-1 levels in lung tissues detected by Real-time PCR and Western blot analysis (**Supplementary Figures 1A, D**). Moreover, CD206—a surface marker of M2 macrophages—was significantly elevated in MS_Rv3737-infected macrophages at 12- and 24-hours post-infection, as shown by increased MFI levels (**Figures 3F, G**). These results suggest that Rv3737 promotes M2 macrophage polarization and suppresses the M1-associated pro-inflammatory response.

### 3.4 Rv3737 facilitated mycobacteria survival and aggravated lung pathology progression *in vivo*

**Figure 4:**
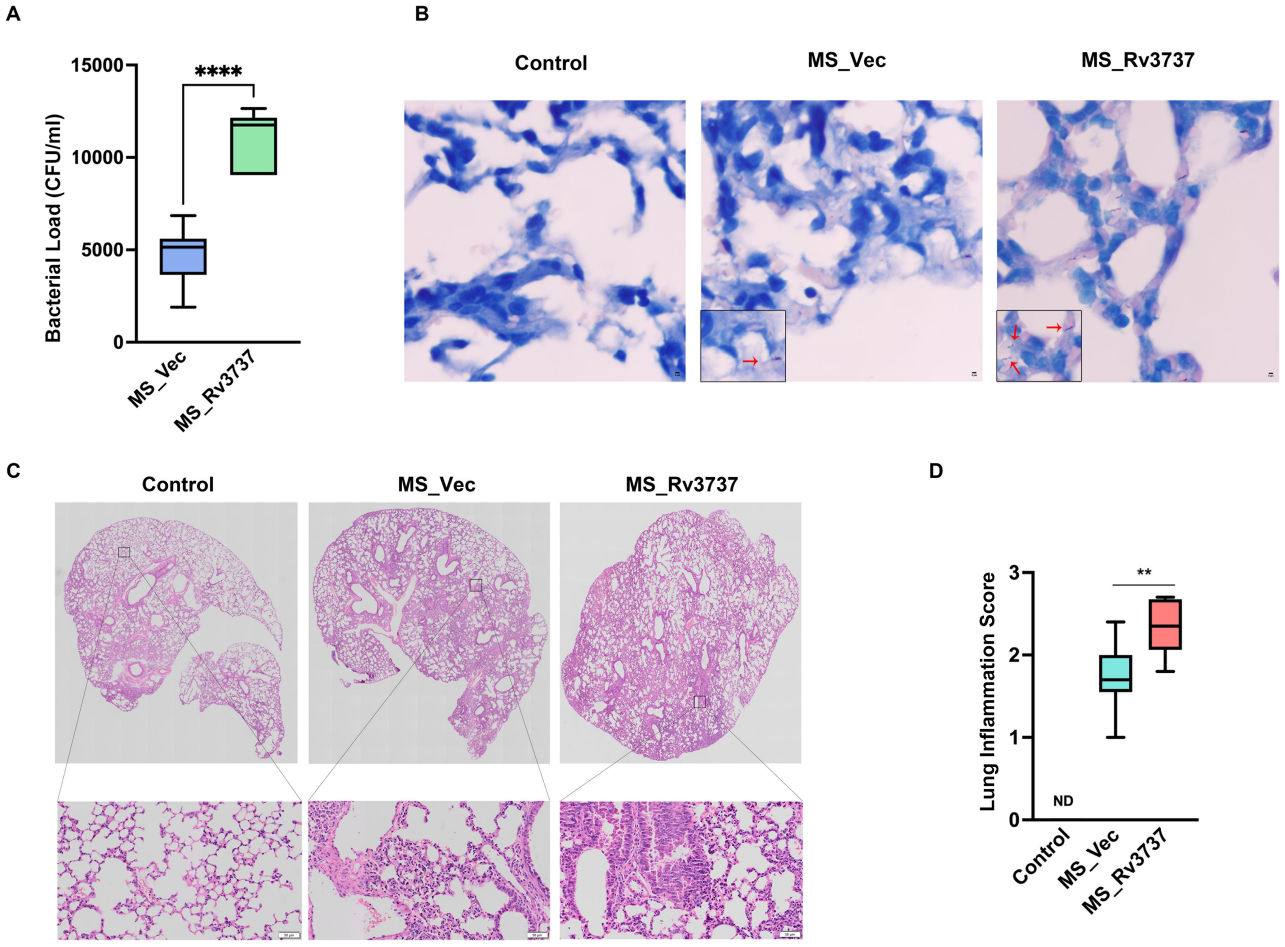
Rv3737 facilitated mycobacteria survival and aggravated lung pathology progression in MS-infected mice. 6–8-week C57BL/6 mouse was infected via nasal instillation with Mycobacterium smegmatis at a dose of 2×10^7^/mice. The lung tissues were harvested at day 6 post-infection for colony-forming unit (CFU) analysis, Acid-fast staining, and hematoxylin and eosin (HE). **(A)** CFU in the MS_Vec and MS_Rv3737-infected mice lung; **(B)** Acid-Fast staining in the lungs of MS_Vec or MS_Rv3737-infected mice, red arrow represents acid-fast staining bacilli; **(C)** HE staining in lungs of MS_Vec and MS_Rv3737-infected mice; **(D)** Lung injury scores of infected mice at day 6 postinfection. N.D., not detected. The black square indicates cellular infiltration. Scale bar: 50 μm. Statistical analyses were performed in **(A)** using One way-ANOVA. The data presented are 5–95 percentiles. **p < 0.01, ****p < 0.0001

M2 macrophages promote *M. tuberculosis* survival and dampen host resistance. To further investigate the role of Rv3737 in modulating host defense during Mycobacteria infection *in vivo*, we used the MS_Vec and MS_Rv3737 infected C57BL/6 mice. We established an MS-infected mouse model. On day 6 post-infection, lung bacillary loads were significantly higher in MS_Rv3737-infected mice compared to MS_Vec-infected group (**Figure 4A**). Consistent with these findings, the acid-fast staining data also demonstrated that Rv3737 promoted the mycobacterial survival *in vivo* (**Figure 4B**). Hematoxylin and eosin staining results showed that MS_Rv3737-infected mice obviously aggravated lung pathology progression, as evidenced by inflammatory cells infiltration (**Figures 4C, D**). Overall, these data demonstrated that Rv3737 indeed promoted mycobacterial survival *in vivo* and subsequently exacerbated lung pathology progression *in vivo* by regulating M2-type macrophages polarization.

### 3.5 Deletion of Rv3737 activates NF-κB and MAPK signaling via enhanced phosphorylation of P65 and P38

**Figure 5:**
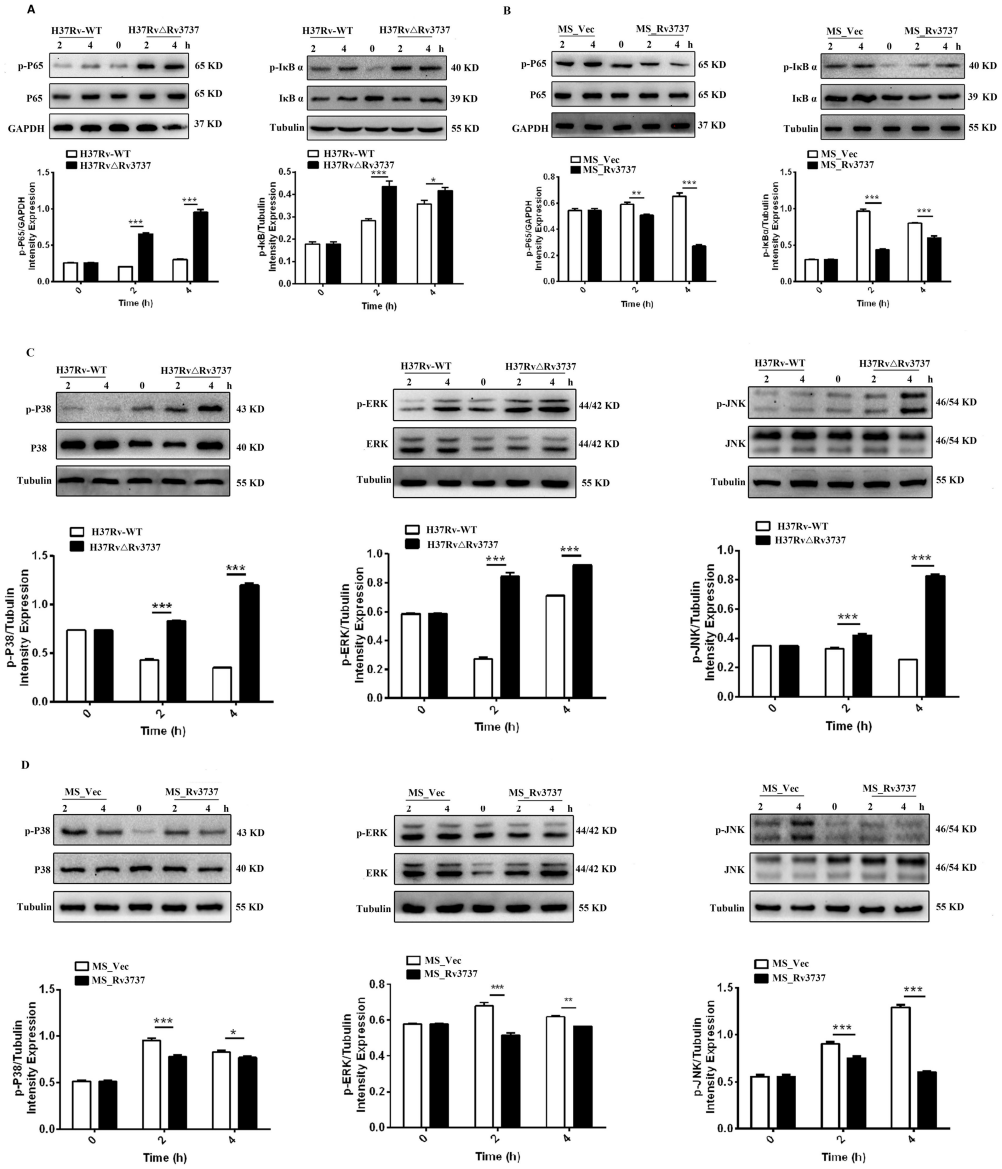
Rv3737 knockout activates NF-κB and MAPK pathways via P65 and P38 phosphorylation. RAW264.7 cells were infected at an MOI of 10. Cells were lysed at 2- and 4-hours post-infection. **(A, B)** Western blot analysis of P65 and IκBα phosphorylation in the NF-κB pathway. **(C, D)** Western blot analysis of phosphorylated P38, JNK, and ERK in the MAPK pathway. *p < 0.05, **p < 0.01, ***p < 0.001.

To investigate the mechanisms by which Rv3737 modulates macrophage polarization, the NF-κB and MAPK signaling pathways are central to regulating macrophage polarization and inflammatory responses. we performed RNA sequencing (RNA-seq) on RAW264.7 macrophages infected with either H37Rv-WT or H37RvΔRv3737. KEGG pathway enrichment analysis of differentially expressed genes revealed significant enrichment of inflammatory pathways, particularly the NF-κB and TNF signaling pathways (**Supplementary Figure 2A**). Consistent with this, mRNA levels of AP-1 and NF-κB were upregulated in H37RvΔRv3737-infected macrophages (**Supplementary Figure 2B**), whereas expression of these transcription factors was significantly suppressed in MS_Rv3737-infected cells (**Supplementary Figure 2C**). To validate these findings, we assessed the phosphorylation status of key signaling molecules by Western blot. In H37RvΔRv3737-infected macrophages, phosphorylation of P65 and IκBα was markedly increased, particularly at 4 hours post-infection (**Figure 5A**), indicating activation of the NF-κB pathway. Conversely, MS_Rv3737-infected cells exhibited reduced phosphorylation of these proteins (**Figure 5B**). We also examined MAPK pathway components. Phosphorylation of P38, JNK, and ERK was significantly elevated in H37RvΔRv3737-infected cells, with P38 showing the most pronounced increase (**Figure 5C**). In contrast, MS_Rv3737 overexpression led to decreased phosphorylation of these MAPKs compared to vector control (**Figure 5D**). Collectively, these results indicate that deletion of Rv3737 activates the P65/NF-κB and P38/MAPK pathways, suggesting their involvement in Rv3737-mediated regulation of macrophage polarization.

### 3.6 Rv3737 deletion promotes M1 polarization and antimicrobial activity via the P65/NF-κB and P38/MAPK pathways

**Figure 6:**
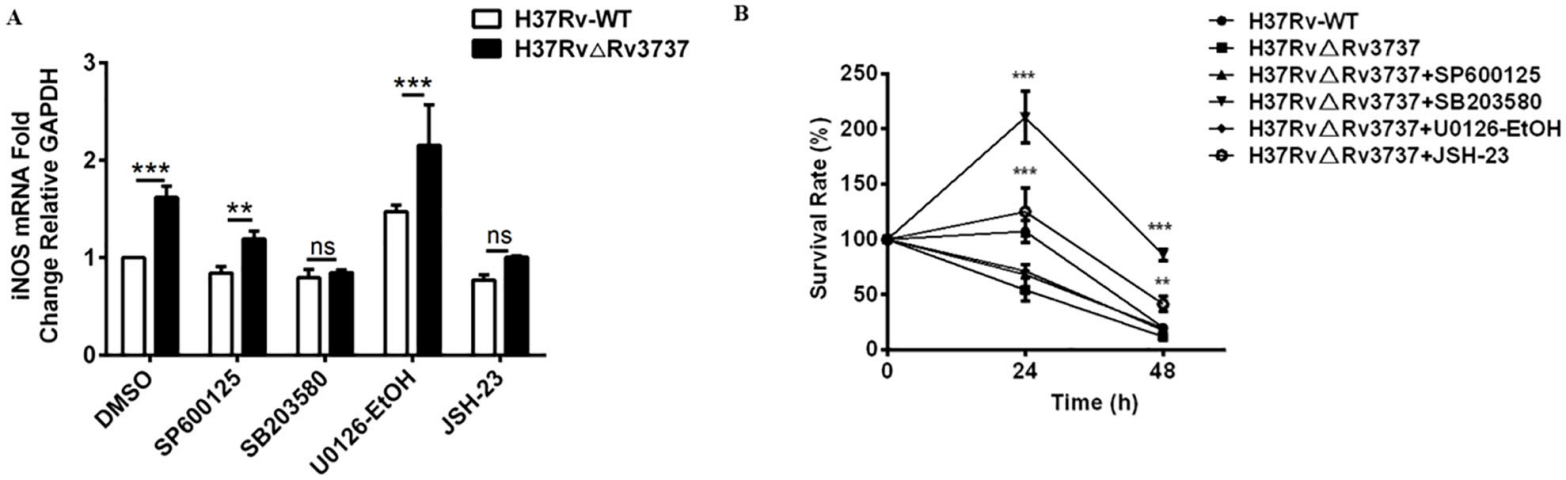
Rv3737 deletion enhances M1 polarization and antimicrobial activity via P65/NF-κB and P38/MAPK signaling. RAW264.7 cells were pre-treated with SP600125 (JNK inhibitor), SB203580 (P38 inhibitor), U0126-EtOH (ERK inhibitor), or JSH-23 (P65 inhibitor) before infection with H37Rv-WT or H37Rv△Rv3737. **(A)** iNOS mRNA levels determined by real-time PCR. **(B)** CFU assay for intracellular bacterial survival at 0-, 24-, and 48-hours post-infection; CFUs were counted after incubation on 7H10 agar for 3–4 weeks. **p < 0.01, ***p < 0.001. ns, no significant.

To determine whether the activation of NF-κB and MAPK signaling mediates the enhanced M1 polarization and bactericidal activity observed in Rv3737-deficient macrophages, we pretreated RAW264.7 cells with specific inhibitors targeting JNK (SP600125), P38 (SB203580), ERK (U0126-EtOH), and P65/NF-κB (JSH-23). As expected, iNOS mRNA expression was significantly upregulated in H37RvΔRv3737-infected macrophages. However, this increase was markedly reversed by pretreatment with P38 and P65 inhibitors, whereas inhibition of JNK or ERK had minimal effects (**Figure 6A**). Furthermore, the enhanced antimicrobial activity associated with H37RvΔRv3737 infection was diminished upon P38 or P65 pathway inhibition, as evidenced by increased intracellular bacterial survival (**Figure 6B**). These results suggest that Rv3737 knockout promotes M1-type macrophage polarization and enhances the antimicrobial response by activating the P65/NF-κB and P38/MAPK signaling pathways, thereby reducing intracellular Mtb survival (**Figure 6B**).

## 4 Discussion

Tuberculosis (TB), caused by *Mycobacterium tuberculosis* (Mtb), remains a major global health concern. Mtb has evolved sophisticated mechanisms to survive within macrophages, primarily by subverting host immune responses, including the regulation of macrophage polarization. During M1 polarization, macrophages mount antimicrobial responses characterized by the production of nitric oxide (NO), enhanced antigen presentation, and secretion of pro-inflammatory cytokines—key processes critical for controlling intracellular pathogens like Mtb (Liu et al., 2022). Several Mtb genes, including those encoding transmembrane proteins such as Rv0309 (Peng et al., 2022), Rv0426c (Ruan et al., 2020), and PknF (Rastogi et al., 2021), have been implicated in modulating macrophage polarization. However, the function of Rv3737, another putative transmembrane protein, has remained unclear. In this study, we explored its role in macrophage polarization and immune signaling, offering new insights into host-pathogen interactions at the molecular level.

Upon infection, Mtb enters macrophages via receptor-ligand interactions, triggering phagocytosis and enabling the bacterium to establish intracellular survival niches (Chen et al., 2023). These interactions are mediated by membrane-associated proteins from both the host and pathogen. The Mtb cell envelope harbors numerous membrane proteins with enzymatic, structural, and virulence-related functions that contribute to immune modulation and survival. Our bioinformatic analysis revealed that Rv3737 is a transmembrane protein with 10 membrane-spanning segments and a high content of aliphatic amino acids—features consistent with roles in host interaction, membrane localization, and immune modulation. These characteristics are reminiscent of other virulence-associated membrane proteins, such as components of the ESX-1 secretion system, which are critical for Mtb pathogenicity (Roy et al., 2020).

Beyond transmembrane proteins, Mtb secretory and cell wall-associated proteins are known to shape macrophage responses. Rv3737 encodes a 55 kDa protein homologous to ThrE, a member of a novel transporter family linked to amino acid export and intracellular survival (Li et al., 2022). In our study, Rv3737 deletion led to increased expression of M1 markers—including TNF-α, IL-6, iNOS, IL-1β, MCP-1, IL-12b, and CD86—while M2 markers were significantly suppressed. This is consistent with previous reports indicating that M1 macrophages drive bactericidal activity through heightened inflammatory responses (Russell et al., 2025). Other Mtb proteins also manipulate macrophage phenotypes. For example, ESAT-6 and CFP-10 promote M2 polarization, while ESAT-6 also exhibits M1-inducing effects during early infection (Deng et al., 2023). Proteins such as HSP16.3 shift macrophages from an M1 to M2 state, and PPE36 promotes Mtb persistence by suppressing M1 polarization (Zhang et al., 2020; Gong et al., 2021). Similarly, Rv0426c impairs macrophage apoptosis, facilitating bacterial replication (Ruan et al., 2020). Our findings suggest that Rv3737 deletion disrupts this immune evasion strategy by promoting M1-type polarization and inhibiting M2 responses, potentially enhancing the host’s antimicrobial defense.

The mechanism of macrophage polarization is complex and involves multiple signaling pathways. Our RNA sequencing (RNA-seq) analysis revealed that deletion of Rv3737 in *M. tuberculosis* significantly enriched gene expression in the NF-κB and TNF-α signaling pathways. The NF-κB and MAPK signaling cascades are well-established as central regulators of inflammation and M1-type macrophage polarization. Consistent with these findings, our results showed that Rv3737 deletion markedly enhanced the phosphorylation of P65 and P38, key indicators of activation in these pathways. Furthermore, pharmacological inhibition of P65 (via JSH-23) and P38 (via SB203580) reversed the upregulation of the M1 macrophage marker iNOS and led to a significant increase in bacterial load within macrophages. These results support the notion that Rv3737 regulates M1 macrophage polarization via the NF-κB and P38/MAPK signaling pathways. Previous studies reinforce this concept. For example, the Mtb transmembrane protein Rv0309 modulates macrophage polarization by targeting MAPK and NF-κB signaling, thereby suppressing M1 responses and promoting bacterial survival (Peng et al., 2022). Similarly, Rv2626c activates NF-κB signaling through direct interaction with TLR2, leading to downstream effects on macrophage polarization (Kim et al., 2020). Moreover, the Mtb protein PPE36, which also contains a transmembrane domain, has been shown to inhibit M1 polarization through the ERK branch of the MAPK pathway (Gong et al., 2021). Together, these findings suggest that Rv3737, like other Mtb transmembrane proteins, influences immune responses by modulating macrophage polarization to favor intracellular persistence. Although the precise mechanism by which Rv3737 engages host intracellular signaling molecules remains unclear, several lines of evidence point toward possible pathways. Rv3737 is a threonine-rich transmembrane protein, and our previous work demonstrated that deletion of Rv3737 in H37Rv (H37RvΔRv3737) leads to elevated threonine concentrations *in vitro*, whereas overexpression of Rv3737 in *M. smegmatis* (MS_Rv3737) significantly suppresses threonine levels. Emerging studies suggest that threonine/serine metabolism is closely linked to immune cell function and can coordinate multiple pathways involved in macrophage polarization (Huang et al., 2024). Therefore, it is plausible that Rv3737 affects host macrophage responses by altering intracellular threonine/serine metabolism, which in turn may influence inflammatory signaling pathways such as NF-κB and MAPKs. Moreover, whether Rv3737 exerts its effects through upstream serine/threonine kinases, including the PI3K/mTOR axis, which also regulates NF-κB and MAPKs, remains an open question. Further investigation is needed to explore this potential regulatory link. Although we have not yet identified direct molecular interactions between Rv3737 and specific host signaling proteins, our findings lay a foundational framework for future mechanistic studies.

This study also has several limitations. First, due to biosafety constraints, we were unable to perform *in vivo* experiments using H37Rv-infected mouse models. Alternatively, we evaluated the M1 and M2 macrophage polarization markers iNOS and Arg1 in MS_Rv3737 and MS_Vec-infected mice; the results were consistent with *in vitro* findings. These findings further provide *in vivo* support for the immunomodulatory effects of Rv3737 observed *in vitro*. Moreover, the precise mechanisms by which Rv3737 influences the NF-κB and MAPK signaling pathways remain to be elucidated. Future studies will focus on identifying potential interacting partners or investigating whether Rv3737 modulates these pathways through the regulation of threonine metabolism.

In conclusion, our findings demonstrate that deletion of Rv3737 promotes M1 macrophage polarization and reduces *M. tuberculosis* survival in macrophages by activating the P65 and P38 signaling pathways. This study offers new insights into the role of Rv3737 in modulating immune signaling and macrophage polarization, thereby enhancing our understanding of Mtb’s immune evasion strategies and highlighting potential targets for therapeutic intervention.

## 5 Conclusion

Deletion of Rv3737 in *M. tuberculosis* H37Rv markedly increased the expression of M1 macrophage polarization markers (CD86, iNOS, IL-1β, IL-6, and MCP-1), while significantly reducing the expression of M2 markers (Arg-1, IL-10, TGF-β, and CD206). Since M1 macrophages exert potent bactericidal activity through activation of the NF-κB and P38 MAPK pathways, these findings highlight the role of Rv3737 in modulating host immune responses. Overall, this study advances our understanding of macrophage polarization in tuberculosis and provides a potential foundation for the development of host-directed therapies against TB.

## Data Availability

The original contributions presented in the study are included in the article/**Supplementary Material**. Further inquiries can be directed to the corresponding authors.

## Supplementary material

Supplementary Figure 1 Rv3737 promoted Arg1 expression *in vivo*. 6–8-week C57BL/6 mouse was infected through nasal instillation with Mycobacterium smegmatis at 2×10^7^/mice, the lung tissues were harvested at day 6 post-infection for cytokine measurement, and Western blot analysis. **(A, C)** Real-time PCR detected the Arg1 and iNOS mRNA expression; **(B)** Western blot analysis of Arg1 and iNOS expression in MS_Vec or MS_Rv3737-infected mice; **(D, E)** Arg1 and iNOS intensity relative to Tubulin.

Supplementary Figure 2 Rv3737 Deletion Enhances NF-κB and AP-1 Expression, Promoting M1 Polarization. **(A)** KEGG pathway enrichment analysis of top 20 enriched pathways in RAW264.7 cells infected with H37Rv-WT or H37Rv△Rv3737 for 24 hours. **(B, C)** Real-time PCR analysis of macrophage polarization-related signaling molecules (STAT1, AP-1, IRF3, IRF5, JAK1, JAK2, NF-κB, C/EBPβ, CREB, STAT2, STAT5, TAT6, IRF4, JAK3, JMJD3, PI3K, PPARγ) in cells infected with H37Rv-WT, H37Rv△Rv3737, MS_Vec, or MS_Rv3737. *p < 0.05, **p < 0.01, ***p < 0.001.

Supplementary Table 1 Primer sequences used for detecting M1/M2 polarization-associated cytokines and macrophage transcription factors by real-time PCR.
