# Supplementary figures and images for: Depletion of Mycobacterium tuberculosis transmembrane protein Rv3737 reduces pathogen survival and induces M1 macrophage polarization against tuberculosis
Source: Front Cell Infect Microbiol. 2025 Sep 2;15:1592296. doi: 10.3389/fcimb.2025.1592296 (PMC12436412; doi:10.3389/fcimb.2025.1592296)

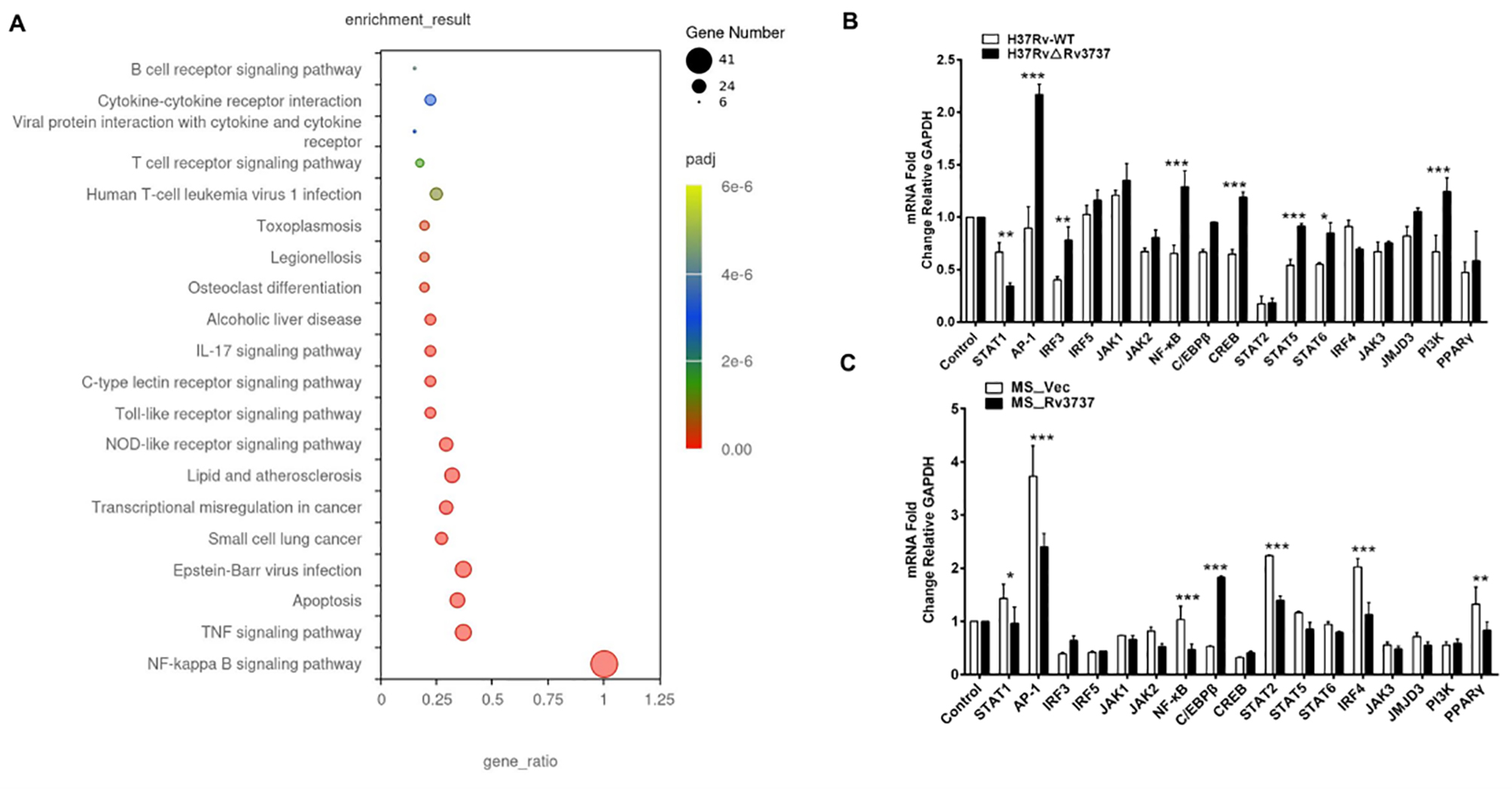

Supplement: Supplementary Figure 2 — Rv3737 Deletion Enhances NF-κB and AP-1 Expression, Promoting M1 Polarization. (A) KEGG pathway enrichment analysis of top 20 enriched pathways in RAW264.7 cells infected with H37Rv-WT or H37Rv△Rv3737 for 24 hours. (B, C) Real-time PCR analysis of macrophage polarization-related signaling molecules (STAT1, AP-1, IRF3, IRF5, JAK1, JAK2, NF-κB, C/EBPβ, CREB, STAT2, STAT5, TAT6, IRF4, JAK3, JMJD3, PI3K, PPARγ) in cells infected with H37Rv-WT, H37Rv△Rv3737, MS_Vec, or MS_Rv3737. *p < 0.05, **p < 0.01, ***p < 0.001. [file Image2.jpeg]
